# ATM Inhibitor Suppresses Gemcitabine-Resistant BTC Growth in a Polymerase θ Deficiency-Dependent Manner

**DOI:** 10.3390/biom10111529

**Published:** 2020-11-09

**Authors:** Yi-Ru Pan, Chiao-En Wu, Chun-Nan Yeh

**Affiliations:** 1 Department of General Surgery and Liver Research Center, Chang Gung Memorial Hospital, Linkou branch, Chang Gung University, Taoyuan 333, Taiwan; panyiru0331@gmail.com; 2 Division of Hematology-Oncology, Department of Internal Medicine, Chang Gung Memorial Hospital, Linkou, Chang Gung University College of Medicine, Taoyuan 333, Taiwan; jiaoen@gmail.com

**Keywords:** ATM, DNA polymerase θ, gemcitabine-resistance, biliary tract cancer

## Abstract

Patients with advanced biliary tract cancer (BTC) inevitably experience progression after first-line, gemcitabine-based chemotherapy, due to chemo-resistance. The genetic alterations of DNA damage repair (DDR) genes are usually determined in BTC tumors. In this study, we found that the *POLQ* mRNA levels are downregulated and the ataxia-telangiectasia mutated (ATM) inhibitor AZD0156 was more sensitive in gemcitabine-resistant BTC sublines than in the parental cell lines. The knockdown of DNA polymerase θ does not affect cell proliferation, but its combination with the ATM inhibitor facilitated cell death in gemcitabine-resistant and gemcitabine-intensive BTC cells. Moreover, in the DNA damage caused by photon, hydrogen peroxide, or chemotherapy drugs, synthetic lethal interactions were found in combination with ATM inhibition by AZD0156 and DNA polymerase θ depletion, resulting in increased DNA damage accumulation and micronucleus formation, as well as reduced cell survival and colony formation. Collectively, our results reveal that ATM acts as a potential target in gemcitabine-resistant and DNA polymerase θ-deficient BTC.

## 1. Introduction

For patients with advanced biliary tract cancer (BTC), cisplatin (CDDP) and gemcitabine (GEM) are typically used as the first-line chemotherapy. However, not all the patients obtain benefits from gemcitabine-based chemotherapy, due to primary and second resistance [1]. The molecular pathogenesis in cholangiocarcinoma (CCA) displays multiple genomic alterations, such as *TP53*, *KRAS,* and *IDH* [2]. Among them are several genes with genomic alterations involved in DNA damage repair (DDR) systems, such as p53, KMT2C (lysine methyltransferase 2C), ATM (ataxia-telangiectasia mutated), and ATR (ataxia telangiectasia and Rad3-related). DNA damage repair (DDR) deficiency is usually detected in different cancers, including BTC [1]. In previous studies, about 25% of BTC patients were found to have DDR deficiency [1]. Thus, the development of targeted therapies for DDR deficiency has been considered for clinical trials on BTC.

DNA damage occurs every day in our cells, including single-strand breaks (SSBs), double-strand breaks (DSBs), bulky adducts, base alkylation, base mismatches, insertions, and deletions [3]. There are three possible mechanisms of DNA repair that can ameliorate DSBs [4]. Homologous recombination (HR) repair is the major repair pathway for DSBs in the late S to G2 phases, and canonical, non-homologous end joining (c-NHEJ) operates predominantly in the G1 phase [5]. The third pathway of DNA repair for DSBs is the alternative non-homologous end joining (alt-NHEJ) pathway, suppressed by c-NHEJ repair and HR repair [6]. ATM is activated during DSB damage and plays a crucial role in DNA repair [7]. ATM is recruited to DSB sites by the MRE11–RAD50–NBS1 (MRN) complex, leading to the phosphorylation of histone H2AX Ser139 (γ-H2AX) and phosphorylating several important molecules, such as 53BP1 (p53-binding protein 1) and BRCA1 (breast cancer 1) for DNA repair [8,9].

DNA polymerase θ, encoded by *POLQ*, is an enzyme that is involved in alternative, non-homologous end-joining (alt-NHEJ). The C-terminal is the family A of DNA polymerases, with additional insertion elements whose N-terminal is a helicase-like domain with DNA-dependent ATPase activity [6]. DNA polymerase θ has been demonstrated to suppress homologous recombination (HR) repair via associating with RAD51 [10]. In previous reports, the knockout of POLQ caused synthetic lethality in BRCA1 mutant cells and FANCD2-defective human cells [10,11]. A combination of *atm* deficiency and *polq* loss-of-function background caused synthetic semi-lethality, and the few surviving mice displayed increased chromosomal instability [12].

Synthetic lethality indicates cell death caused by the deficiencies of two genes. The alteration of a single gene does not lead to cell death, but the additional deficiency of the second gene leads to a loss of viability [13]. Genomic alterations are a common cause of drug resistance [14], and synthetic lethality approaches are useful for targeting the tumors with genomic alterations [15].

In this study, we investigated the impact of genomic alterations involved in DDR on gemcitabine resistance. We showed that *POLQ* is downregulated in gemcitabine-resistant BTC cells. The increased sensitivity of the ATM inhibitor (AZD0156) was observed in gemcitabine-resistant cells, and AZD0156 alone enhanced cell death in DNA polymerase θ knockdown cells. These observations provide valuable evidence of novel therapeutic strategies for advanced BTC patients.

## 2. Research Design and Methods

### 2.1. Cells and Reagents

BTC cell lines (RBE, HuCCT1, SSP-25, TGBC-24TKB, and TFK-1) were purchased from the RIKEN Cell Bank (Ibaraki, Japan). BTC cell lines (SNU-308 and SNU-1196) were purchased from the Korean cell line bank (Seoul, Korea). RBE, HuCCT1, SSP-25, TFK-1 SNU-308, and SNU-1196 cell lines were grown in RPMI (Roswell Park Memorial Institute) medium supplemented with 10% fetal bovine serum (FBS), penicillin-streptomycin, L-glutamine, and sodium pyruvate. TGBC-24TKB cells were grown in DMEM supplemented with 10% FBS and penicillin–streptomycin. The gemcitabine-resistant cells (SNU-1196-GR and SSP-25-GR cells) were generated in the cell culture system. The cells were treated with a half-maximal inhibitory concentration (IC_50_) dose of gemcitabine for one month (20 nM for SSP-25 cells and 10 nM for SNU-1196 cells). After one month, the media were replaced with the fresh media containing an IC_90_ dose of gemcitabine (250 nM for SSP-25 cells and 60 nM for SNU-1196 cells) for another month. After two months, the live cells were identified as gemcitabine-resistant cells. All the cell lines used in this study were tested for mycoplasma contamination and authenticated by STR (short tandem repeat) method. AZD0156 (ATM inhibitor), gemcitabine, and cisplatin were purchased from AdooQ BioScience (Irvine, CA, USA).

### 2.2. Virus Production and Infection

The pCMV-Δ8.9, pMDG, (from the National RNAi Core Facility, Academia Sinica, Taiwan), and pLKO.1-shRNA (short hairpin RNA) clones were co-transfected into HEK293T cells using jetPEI (Polyplus Transfection, New York, NY, USA) overnight to package the virus. Then, the virus-containing supernatant was collected at 48 and 72 h after transfection, and lentivirus particles were collected and stored at −80 °C. For viral infection, the cells were mixed with virus-containing supernatants supplemented with 8 μg/mL polybrene (Sigma-Aldrich, St. Louis, MO, USA). After infection with the virus for 24 h, the infected cells were selected with 1~1.5 μg/mL puromycin for another three days. The target sequences are listed below: LacZ (5′-TGTTCGCATTATCCGAACCAT-3′), POLQ #1 (5′-CCTTCAATCTTGCTTGCGAAA-3′), and POLQ #2 (5′-CGGGCCTCTTTAGATATAAAT-3′).

### 2.3. Analysis of DDR Gene Expression

The POLQ expression of GEM-resistant cell lines (SSP-25-GR and SNU-1196-GR) was analyzed using the Affymetrix Human Genome U133A Array (Thermo Fisher Scientific, Inc., Waltham, MA, USA). The expression of DDR-related genes was obtained from The Cancer Genome Atlas (TCGA) and analyzed via starBase (http://starbase.sysu.edu.cn/index.php). The survival analysis was performed by UCSC Xena (https://xena.ucsc.edu/).

### 2.4. RNA Extraction and Quantitative RT-PCR (RT-qPCR)

RNA was extracted using Trizol, and 1 μg RNA was used for reverse transcription with a HiScript I First Strand cDNA Synthesis Kit (Bionovas, Taipei, Taiwan), according to the manufacturer’s instructions. Next, qPCR was performed using 1 μL of cDNA. The qPCR reaction mixtures (20 μL) contained 10 µl of Fast SYBR Green Master Mix (Thermo Fisher Scientific, Inc., Waltham, MA, USA) and 0.5 µM forward and reverse primers. After an initial denaturation cycle (95 °C for 20 s), the product was amplified at 95 °C for 3 s and 60 °C for 30 s for 40 PCR cycles, using an Applied Biosystems QuantStudio 5 Real-Time PCR System (Thermo Fisher Scientific, Inc., Waltham, MA, USA). The primers for qPCR are listed below: POLQ-F (5′-GACCAAACAGGATTGTCACGA-3′), POLQ-R (5′-GCTGGCGCCTATTTTCACTT-3′), GAPDH-F (5′-GTCTCCTCTGACTTCAACAGCG-3′), GAPDH-R (5′-ACCACCCTGTTGCTGTAGCCAA-3′).

### 2.5. Cytotoxicity, Cell Proliferation, and Cell Viability Assays

**Figure 1:**
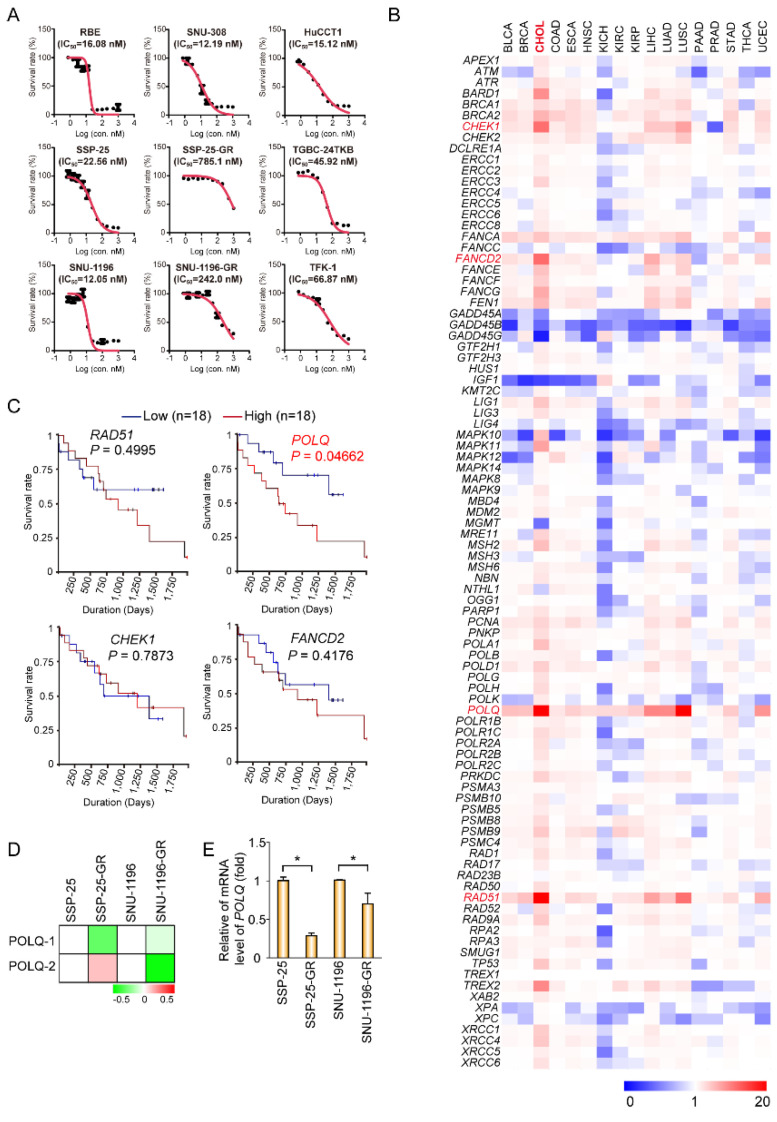
DNA polymerase θ may be involved in gemcitabine resistance in BTC (biliary tract cancer) cells. (**A**) Cell viability in various concentrations of GEM (gemcitabine). The values (means ± SD) are from two data points and are expressed as a percentage relative to that of the cells without gemcitabine treatment. The IC_50_ (half-maximal inhibitory concentration) values for gemcitabine in BTC cell lines are shown in each panel. (**B**) A heat map showing the relative mRNA expression of 92 DNA damage repair (DDR) genes in 17 different cancers from cancer tissues, compared to their normal tissues. The data were obtained from The Cancer Genome Atlas (TCGA) and analyzed by starBase (http://starbase.sysu.edu.cn/index.php). Red pixels indicate upregulated expression; blue pixels represent downregulated expression. (**C**) The data of the Kaplan–Meier survival curves for overall survival in 36 patients with cholangiocarcinoma were obtained from TCGA and analyzed by UCSC Xena (https://xena.ucsc.edu/). The *p* values are shown in each panel. (**D**) A heat map showing the relative mRNA expression of POLQ using different probes (POLQ-1 and POLQ-2) from resistant cells (SSP-25-GR and SNU-1196-GR) compared to their parental cells (SSP-25 and SNU-1196). Red pixels indicate upregulated expression; green pixels represent downregulated expression. (**E**) The relative mRNA level of POLQ. The values (means ± SD) are from two independent experiments, and are presented as the fold change relative to the level of parental cells (SSP-25 and SNU-1196). * *p* < 0.05 by Mann–Whitney U test.

**Figure 2:**
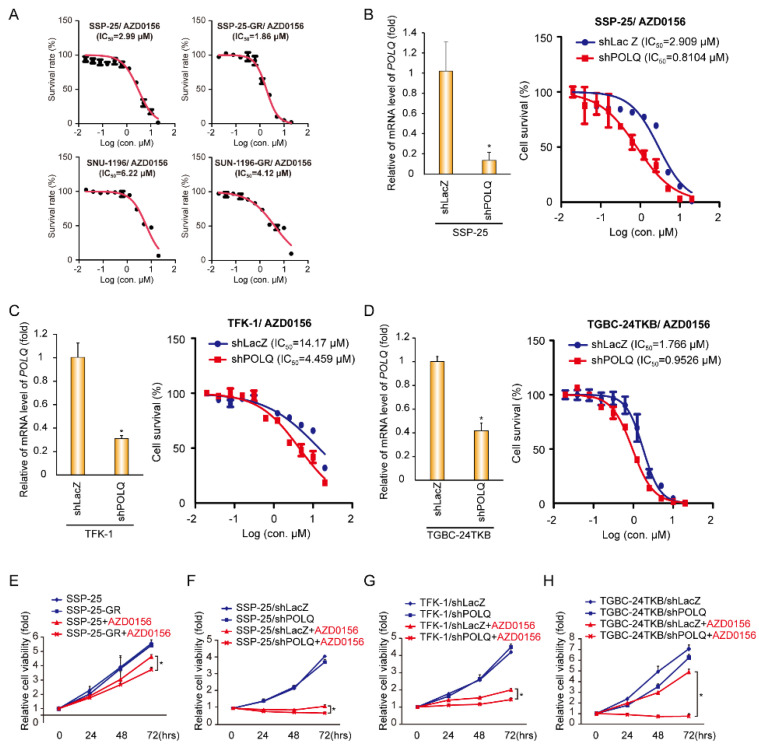
Knockdown of DNA polymerase θ increases AZD0156 sensitivity in BTC cells. (**A**) Cell viability in various concentrations of AZD0156. The values (means ± SD) are from two data points and expressed as a percentage relative to that of the cells without AZD0156 treatment. IC_50_ values for AZD0156 in the BTC cell lines are shown in each panel. (**B**–**D**) DNA polymerase θ was depleted by shRNAs (shPOLQ) in SSP-25 cells (**B**), TFK-1 cells, (**C**) or TGBC-24TKB cells (**D**). Left: the relative mRNA level of POLQ. The values (means ± SD) are from two independent experiments, and are presented as fold-change relative to the level of the cells receiving shRNAs against LacZ (shLacZ). * *p* < 0.05 by Student’s *t*-test. Right: the cell viability in various concentrations of AZD0156. The values (means ± SD) are from two data points, and are expressed as a percentage relative to that of the cells without AZD0156 treatment. The IC_50_ values for AZD0156 in the cell lines are shown in the panel. (**E**) Cell proliferation assay. A total of 5 × 10^3^ cells were seeded in a 96-well plate for 16–24 h, and were then cultured in the absence or presence of 1.25 μM AZD0156 for 0, 24, 48, or 72 h. Cell viability was quantified by CCK-8. The values (means ± SD) are from two independent experiments, and are presented as the fold change relative to the baseline (0 h). * *p* < 0.05 by Student’s *t*-test. (**F**–**H**) Cell proliferation assay. DNA polymerase θ was depleted by shRNAs (shPOLQ) in SSP-25 cells (**F**), TFK-1 cells (**G**), or TGBC-24TKB cells (**H**). A total of 5 × 10^3^ cells were seeded in a 96-well plate for 16–24 h, and were then cultured in the absence or presence of 1.25 μM (F), 5 μM (G), or 1 μM (H) AZD0156 for 0, 24, 48, or 72 h. The cell viability was quantified by CCK-8. The values (means ± SD) are from two independent experiments, and are presented as the fold change relative to the baseline (0 h). * *p* < 0.05 by Student’s *t*-test.

For half-maximal inhibitory concentration (IC_50_) detection, the cells (5 × 10^3^) were seeded in 96-well plates for 16–24 h, and then cultured in various concentrations of GEM or AZD0156. For Figure 1, cell viability was quantified by a CCK-8 (Cell Counting Kit-8) assay (Dojindo Molecular Technologies, Inc., Kumamoto, Japan), according to the manufacturer’s instructions. The concentration of IC_50_ was calculated using Prism. In Figure 2, the cells (5 × 10^3^) were seeded in 96-well plates for 16–24 h, and then cultured in the absence or presence of AZD0156 for 0, 24, 48, or 72 h for cell proliferation and viability. Cell viability was quantified by a CCK-8 assay. As illustrated in Figure 4, 5Gy photon, and 1 μm H_2_O_2_ were added for 2 h, and H_2_O_2_ was washed before the addition of AZD0156. As shown in Figure 6, GEM and CDDP were added for 24 h, and chemotherapy drugs were washed before the addition of AZD0156.

### 2.6. Immunoblotting and Immunofluorescent Staining

For immunoblotting, the cells were lysed in 1% Nonidet P-40 lysis buffer (150 mM NaCl, 1.0% NP-40, 0.5% sodium deoxycholate, 0.1% SDS, 50 mM Tris, pH 7.5) plus protease inhibitors (Roche, Mannheim, Germany), and incubated on ice for at least 30 min. The cell lysates were centrifuged at 13,000 rpm for 10 min at 4 °C, and the protein concentrations were determined via BCA (bicinchoninic acid) protein assays (Thermo Scientific Pierce BCA Protein Assay, Waltham, MA, USA). The proteins were loaded on 6%~12% SDS-PAGE gels for separation with the running buffer, and were then transferred onto PVDF (polyvinylidene difluoride) membranes (Millipore, Billerica, MA, USA). The membranes were blocked with 5% skim milk in TBST (Tris-Buffered Saline and Tween 20) at room temperature for 0.5–2.0 h, and were then incubated with the specific primary antibodies at 4 °C overnight. The membranes were washed with TBST and incubated with secondary antibodies in TBST for 1–2 h at room temperature. The results were observed using UVP ChemStudio PLUS Touch (Analytik Jena AG, Jena, Germany). For immunofluorescent staining, cells were fixed with 4% paraformaldehyde in PBS for 30 min at room temperature, and permeabilized with 0.1% Triton X-100 in PBS for 10 min at room temperature. The cells were blocked with 4% FBS in PBS at room temperature for 2 h, and then were incubated with the specific primary antibodies in 4% FBS at 4 °C overnight. The fixed cells were washed with PBS and incubated with Alexa Fluor Plus 488-conjugated secondary antibodies (Thermo Fisher Scientific, Inc., Waltham, MA, USA) in PBS at 4 °C overnight. Coverslips were mounted in Mounting Medium With DAPI—Aqueous, Fluoroshield (Abcam Plc., Cambridge, United Kingdom) and viewed using a laser-scanning confocal microscope image system (Leica TCS SP8X, Leica Microsystems, Wetzlar, Germany) with a Plan-Apochromat 20×, a Plan-Apochromat 40×, or a Plan-Apochromat 60× objective (Leica). The total fluorescent intensity of γ-H2AX was normalized by the nucleus intensity and quantified from 0.16 mm^2^ per random field. A total of five random fields were measured. The primary antibodies used in this study were mouse monoclonal anti-ATM pS1981 (200-301-400S, Rockland Immunochemicals, Inc., Limerick, PA, USA), mouse monoclonal anti-ATM (clone 2C1, GTX70103, GeneTex, Inc. Alton Pkwy Irvine, CA, USA), mouse monoclonal anti-α-Tubulin (T6793, Sigma-Aldrich, St. Louis, MO, USA) mouse monoclonal anti-phospho-Histone H2A.X Ser139 (clone JBW301, EMD Millipore, Billerica, MA, USA), and rabbit monoclonal anti-PARP1 (poly ADP-ribose polymerase 1; 9532, Cell Signaling Technology, Inc., Danvers, MA, USA).

### 2.7. Comet Assay

The comet assay was performed according to the manufacturer’s instructions (ab238544, Abcam Plc., Cambridge, United Kingdom). Briefly, the TGBC-24TKB cells were seeded in six-well plates for 16–24 h, and then were cultured in 0.5 μM GEM and 5 μM CCDP for 24 h. Before analysis, the cells were cultured in the absence or presence of 5 μM AZD0156 for 4 h. The cells were washed twice with cold Ca^2+^ and Mg^2+^-free PBS, and collected by gentle scraping. A total of 1500 cells in 15 μL were mixed with 60 μL comet agarose. The mixtures were transferred onto the comet slides and allowed to solidify at 4 °C for 30 min. The cells were lysed at 4 °C overnight, and electrophoresis was performed with a cold alkaline electrophoresis buffer for 40 min. The comet slides were rinsed in H_2_O for 5 min three times, and were fixed with 70% cold ethanol for 5 min. The air-dried slides were stained with Vista Green DNA Dye and viewed using a laser-scanning confocal microscope image system (Leica TCS SP8X, Leica Microsystems, Wetzlar, Germany) with a Plan-Apochromat 20× objective (Leica). A total of 50 nuclei were measured.

### 2.8. Micronucleus Assay

The cells were grown on glass coverslips seeded in six-well plates overnight. They were then cultured in media with chemotherapy drugs for 24 h or H_2_O_2_ for 4 h, and cultured in the presence of AZD0156 for 24 h. Next, the media were replaced, and new media containing 3 μg/mL cytochalasin B were added for another 72 h. After 72 h, the cells were fixed with 4% paraformaldehyde in PBS for 30 min at room temperature, permeabilized with 0.1% Triton X-100 in PBS for 10 min at room temperature, and the nuclei were stained using Mounting Medium with DAPI–Aqueous, Fluoroshield (Abcam Plc., Cambridge, United Kingdom). A total of 100 binuclei were measured.

### 2.9. Colony Formation Assay

TGBC-24TKB cells (400 cells/well) were seeded in six-well plates for 14 days. After 14 days, the cells were cultured in the absence or presence of 0.5 μM GEM and 5 μM CCDP for 24 h, and then were cultured in the absence or presence of 1 μM AZD0156 for another 7 days. The cells were then fixed with cold methanol for 10 min and stained with 0.5% crystal violet for 10 min. Next, the six-well plates were washed with water, and the numbers of colonies were measured.

### 2.10. Statistics

The results are presented as means ± SD. A two-tailed, independent Student’s *t*-test or Mann–Whitney U test was used to compare the continuous variables between the two groups. Differences were considered to be statistically significant at *p* < 0.05 for all of the tests.

## 3. Results

### 3.1. The ATM Inhibitor (AZD0156) Decreases Tumor Survival in Gemcitabine-Resistant or DNA Polymerase θ-Depleted BTC Cells

To resolve the problem of failure in first-line chemotherapy, gemcitabine (GEM)-resistant BTC cell lines were established. We first incubated the BTC cell lines SSP-25 and SNU1196 in GEM-containing media for 2 months. Two GEM-resistant BTC sublines were generated (SSP-25-GR and SNU1196-GR). The GEM IC_50_ values were determined in GEM-resistant sublines and several BTC cell lines. The IC_50_ of GEM in SSP-25-GR was increased 30 times compared to the parental SSP-25 cells (22.56 nM for SSP-25 cells, 785.1 nM for SSP-25-GR cells). The IC_50_ value in SNU-1196-GR was increased 20 times compared to the parental SNU-1196 cells (12.05 nM for SNU-1196 cells; 242.0 nM for SNU-1196-GR cells) (Figure 1A). The inhibitions of some DDR molecules, such as CHK1 (checkpoint kinase 1) and ATR, have been demonstrated to enhance the effect of GEM in resistant pancreatic cancers [16,17]. Thus, targeting DDR molecules may be a new approach for GEM-resistant BTCs. To identify which DDR molecules are potential targets in BTC, the expressions of several DDR-related molecules were analyzed in BTC and control normal tissue using the TCGA database (Figure 1B). Among DNA repair genes, the mRNA expression of some genes (*RAD51*, *POLQ*, *FANCD2*, and *CHEK1*) in CCA was more than tenfold than that in normal tissues (Figure 1B). Moreover, only *POLQ* expression was inversely correlated with the survival rate (Figure 1C). DNA polymerase θ encoded by *POLQ* is an enzyme involved in alternative, non-homologous end joining (alt-NHEJ) or micro-homology-mediated end joining (MMEJ) [6]. In a previous report, a combination of *atm*-deficient and *polq* loss-of-function background caused synthetic semi-lethality [12]. In the GEM-resistant SNU-1196-GR subline, *POLQ* mRNA levels were repressed using two specific microarray probes. In the SSP-25-GR subline, *POLQ* mRNA levels were downregulated in one specific microarray probe (Figure 1D). To confirm the results, RT-qPCR was performed. The mRNA level of *POLQ* was suppressed in both GEM-resistant BTC sublines (Figure 1E), suggesting that ATM may be a target in GEM-resistant or GEM-insensitive BTCs.

We further investigated whether ATM is a potential target in GEM-resistant BTCs. We first tested the ATM inhibitor AZD0156 IC_50_ values in parental and GEM-resistant cell lines. The AZD0156 IC_50_ values in GEM-resistant cell lines (SSP25-GR and SNU1196-GR) were reduced compared to those in the parental cells (SSP-25 and SNU1196) (Figure 2A). To further confirm the effect of DNA polymerase θ on ATM inhibitor sensitivity in BTCs, DNA polymerase θ expression was suppressed by shRNAs, and the AZD0156 IC_50_ values were determined in the control (shLacZ) and DNA polymerase θ knockdown (shPOLQ) BTC cells. In SSP-25 cells, the knockdown of DNA polymerase θ decreased AZD0156 IC_50_ (Figure 2B). Moreover, the knockdown of DNA polymerase θ diminished AZD0156 IC_50_ values in GEM-insensitive BTC cell lines TFK-1 and TGBC-24TKB (Figure 2C,D). During tumor growth, ADZ0156 presented a significant reduction in cell proliferation in GEM-resistant SSP-25-GR cells (Figure 2E). The knockdown of DNA polymerase θ did not affect cell proliferation, and the depletion of DNA polymerase θ by shRNAs increased cell death upon AZD0156 treatment in SSP-25, TFK-1, and TGBC-24TKB cells (Figure 2F–H). These results suggest that the suppression of ATM by its inhibitor AZD0156 represses cell survival in polymerase θ-deficient BTC cells.

### 3.2. DNA Damage Enhances the Effect of AZD0156-Mediated Cell Death in DNA Polymerase θ-Depleted BTC Cells

**Figure 3:**
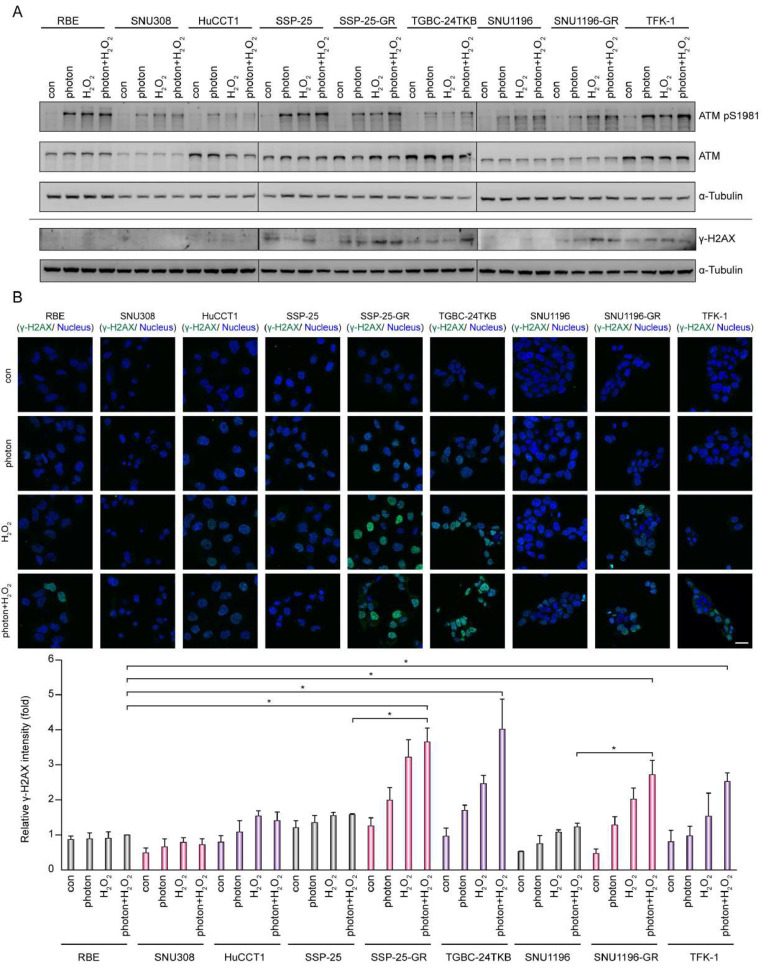
A combination of photons and hydrogen peroxide induces DNA damage in BTC cells. (**A**) Western blots showing the level of histone H2AX Ser139 phosphorylation (γ-H2AX), ataxia-telangiectasia mutated (ATM) Ser1981 phosphorylation, and total ATM in the BTC cell lines receiving 5 Gy photons or 1 μM H_2_O_2_ for 4 h; α-tubulin was the loading control. (**B**) Top: the BTC cells receiving 5 Gy photons or 1 μM H_2_O_2_ for 4 h were fixed and stained with H2AX Ser139 phosphorylation (γ-H2AX) and nuclei. Scale bar = 25 µm. Bottom: the relative fluorescent intensity of γ-H2AX was measured for each field (*n* = 3) and expressed as the fold change relative to the level of RBE cells with the treatment of 5 Gy photons or 1 μM H_2_O_2_ for 4 h. The values (means ± SD) are from three independent experiments. * *p* < 0.05 by Student’s *t*-test.

ATM is activated during DSB damage and plays crucial role in c-NHEJ and HR. DNA polymerase θ participates in the alt-NHEJ repair pathway [6,7]. We used photons or hydrogen peroxide to induce DNA damage and ATM phosphorylation (ATM pS1981), and phosphorylated histone H2AX (γ-H2AX) was shown to increase (Figure 3A,B). The phosphorylated H2AX levels corresponding to DNA damage levels were noted to be higher in two GEM-resistant cell lines, SSP-25-GR and SNU1196-GR, as well as two GEM-insensitive cell lines, TFK-1 and TGBC-24TKB, compared to GEM-sensitive cell lines (Figure 3B), suggesting that more unrepaired DNA damage accumulates in gemcitabine-resistant/insensitive cell lines.

**Figure 4:**
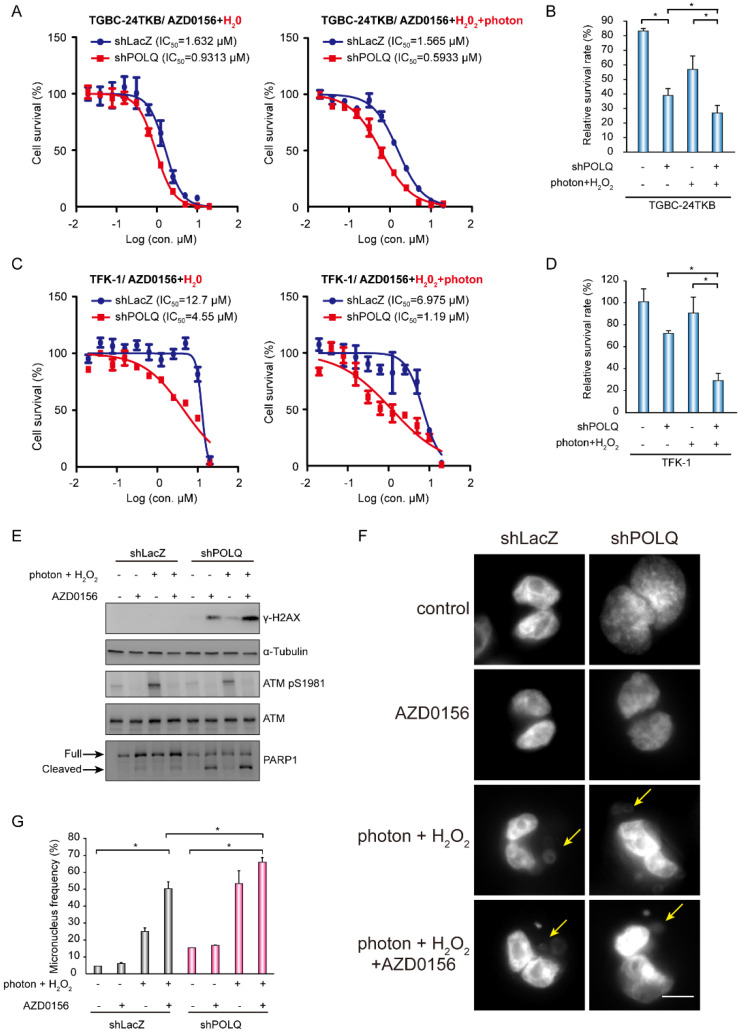
DNA damage enhances the effect of AZD0156-mediated cell death in DNA polymerase θ-depleted BTC cells. (**A**) Cell viability in various concentrations of AZD0156. TGBC-24TKB cells receiving shRNAs specific to DNA polymerase θ (shPOLQ) or LacZ (shLacZ) were treated with 5 Gy photons, and 1 μM H2O2 for 2 h before AZD0156 addition. The values (means ± SD) are from two data points, and are expressed as a percentage relative to that of the cells without AZD0156 treatment. The IC_50_ values for AZD0156 in the cell lines are shown in each panel. (**B**) The relative cell viability in 0.5 μM AZD0156 for 72 h. TGBC-24TKB cells receiving shRNAs specific to DNA polymerase θ (shPOLQ+) or LacZ (shPOLQ–) were treated with 5 Gy photons and 1 μM H_2_O_2_ for 2 h before AZD0156 addition. The values (means ± SD) are from three independent experiments, and are expressed as a percentage relative to that of the cells without AZD0156, 5 Gy photons, and 1 μM H_2_O_2_ treatment; * *p* < 0.05 by Student’s *t*-test. (**C**) Cell viability in various concentrations of AZD0156. TFK-1 cells receiving shRNAs specific to DNA polymerase θ (shPOLQ) or LacZ (shLacZ) were treated with 5 Gy photons and 1 μM H_2_O_2_ for 2 h before AZD0156 addition. The values (means ± SD) are from two data points, and are expressed as a percentage relative to that of the cells without AZD0156 treatment. The IC_50_ values for AZD0156 in the cell lines are shown in each panel. (**D**) The relative cell viability in 2.5 μM AZD0156 for 72 h. TGBC-24TKB cells receiving shRNAs specific to DNA polymerase θ (shPOLQ+) or LacZ (shPOLQ–) were treated with 5 Gy photons and 1 μM H_2_O_2_ for 2 h before AZD0156 addition. The values (means ± SD) are from two independent experiments, and are expressed as a percentage relative to that of the cells without AZD0156, 5 Gy photons, and 1 μM H_2_O_2_ treatment; * *p* < 0.05 by Student’s *t*-test. (**E**) Western blots showing the level of H2AX Ser139 phosphorylation (γ-H2AX), PARP1, ATM Ser1981 phosphorylation, and total ATM in TGBC-24TKB cells receiving shRNAs specific to DNA polymerase θ (shPOLQ) or LacZ (shLacZ). The cells were treated with 5 Gy photons and 1 μM H_2_O_2_ (+) or control H_2_O (–) for 2 h, and then cultured in the absence (–) or presence (+) of 2.5 μM AZD0156 for another 4 h; α-tubulin was the loading control. (**F**,**G**) Micronucleus frequency in TGBC-24TKB cells receiving shRNAs specific to DNA polymerase θ (shPOLQ) or LacZ (shLacZ). The cells were treated with 5 Gy photons and 1 μM H_2_O_2_ (+) or control H_2_O (–) for 2 h, and were then cultured in the absence (–) or presence (+) of 2.5 μM AZD0156 for another 24 h. Representative images of the micronucleus are (arrows) are shown in panel F. The quantitative data are shown in panel G. The values (means ± SD) are from two independent experiments; * *p* < 0.05 by Student’s *t*-test.

We next examined whether the ATM inhibitor AZD0156 enhanced the synergistic effects on cell survival in DNA polymerase θ-deficient BTC cells upon DNA damage. In the presence of photon- and hydrogen peroxide-induced DNA damage, knockdown of DNA polymerase θ decreased AZD0156 IC_50_ values, and most significantly, enhanced cell death in TGBC-24TKB and TFK-1 cells (Figure 4A–D). To further clarify whether the increased cell death upon photon and hydrogen peroxide treatment was caused by DNA damage accumulation, the phosphorylated H2AX levels were assessed. The knockdown of DNA polymerase θ increased phosphorylated H2AX and cleaved PARP1 upon AZD0156 treatment in TGBC-24TKB cells. Notably, with the combination of photons and hydrogen peroxide with AZD0156, phosphorylated H2AX, and cleaved PARP1 were upregulated (Figure 4E). Micronuclei from the damaged DNA were common after cell division. Next, we investigated the effect of DNA polymerase θ knockdown on micronucleus formation. The combination of photons and hydrogen peroxide with AZD0156 significantly promoted the micronucleus frequency in the control or knockdown of DNA polymerase θ TGBC-24TKB cells. The depletion of DNA polymerase θ by shRNAs markedly increased micronucleus formation in the presence of photons and hydrogen peroxide or AZD0156 (Figure 4F,G). Collectively, these data suggest that AZD0156 promotes a significant effect on cell survival in DNA polymerase θ-depleted BTC cells upon photon- and hydrogen-peroxide-induced DNA damage.

### 3.3. A Combination of ATM Inhibition and DNA Polymerase θ Depletion Leads to a Synergistic Effect on Tumor Growth Upon Chemotherapy Drug-Induced DNA Damage in BTC Cells

**Figure 5:**
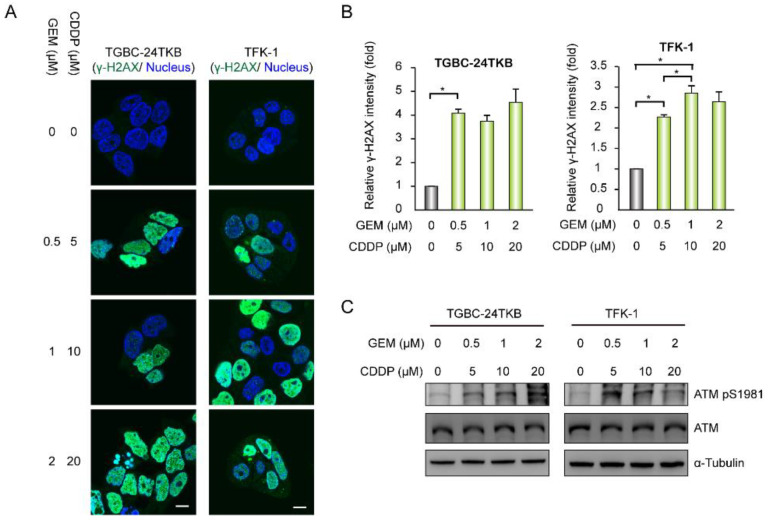
Chemotherapy drugs lead to DNA damage in BTC cells. (**A**) The cells receiving various concentrations of GEM and CDDP for 24 h were fixed and stained with H2AX Ser139 phosphorylation (γ-H2AX) and nuclei. Scale bar = 10 µm. (**B**) The relative fluorescent intensity of γ-H2AX per field was measured (*n* = 5) and expressed as the fold change relative to the levels of the cells without chemotherapy drug treatment. The values (means ± SD) are from two independent experiments; * *p* < 0.05 by Student’s *t*-test. (**C**) Western blots showing the level of ATM Ser1981 phosphorylation and total ATM in the cells receiving various concentrations of GEM and CDDP for 24 h; α-tubulin was the loading control.

Chemotherapy drugs, such as GEM and cisplatin (CDDP) cause DNA damage [18,19]. Thus, we used GEM and CDDP as another approach to induce DNA damage. The formation of γ-H2AX foci and the intensity of γ-H2AX were apparently increased under treatment with different doses of GEM and CDDP (Figure 5A,B). Moreover, the phosphorylated ATM present in the different doses of GEM and CDDP also increased (Figure 5C).

**Figure 6:**
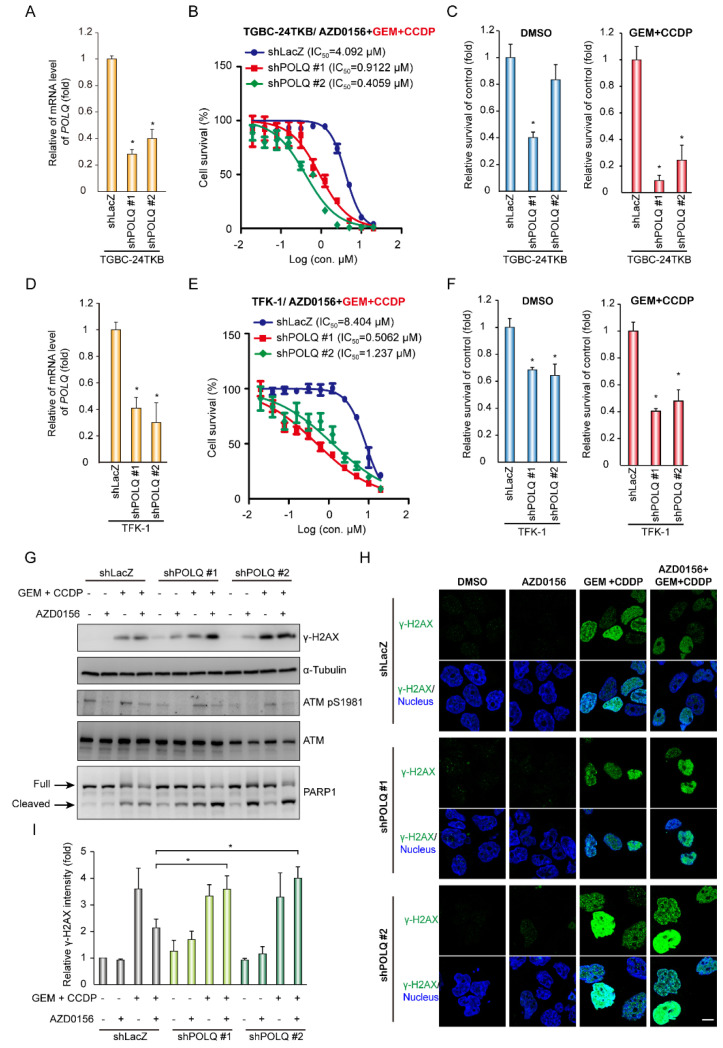
Knockdown of DNA polymerase θ improves AZD0156-mediated cell death. (**A**) DNA polymerase θ was depleted by shRNAs (shPOLQ; #1 and #2) in TGBC-24TKB cells. The relative mRNA level of *POLQ* is shown. The values (means ± SD) are from two independent experiments, and are presented as the fold change relative to the level of the cells receiving shRNAs against LacZ (shLacZ); * *p* < 0.05 by Student’s *t*-test. (**B**) The cell viability in various concentrations of AZD0156. TGBC-24TKB cells receiving shRNAs specific to DNA polymerase θ (shPOLQ; #1 and #2) or LacZ (shLacZ) were treated with 0.5 μM GEM and 5 μM CDDP for 24 h before AZD0156 addition. The values (means ± SD) are from two data points, and are expressed as a percentage relative to that of the cells without AZD0156 treatment. The IC50 values for AZD0156 in the cell lines are shown in each panel. (**C**) The relative cell viability in 0.5 μM AZD0156 for 72 h. TGBC-24TKB cells receiving shRNAs specific to DNA polymerase θ (shPOLQ; #1 and #2) or LacZ (shLacZ) were treated with 0.5 μM GEM and 5 μM CDDP for 24 h before AZD0156 addition. The values (means ± SD) are from three independent experiments, and are expressed as a percentage relative to that of the cells receiving shRNAs against LacZ; * *p* < 0.05 by Student’s *t*-test. (**D**) DNA polymerase θ was depleted by shRNAs (shPOLQ; #1 and #2) in TFK-1 cells. The relative mRNA level of POLQ. The values (means ± SD) are from two independent experiments, and are presented as the fold change relative to the level of the cells receiving shRNAs against LacZ (shLacZ); *p < 0.05 by Student’s *t*-test. (**E**) The cell viability in various concentrations of AZD0156. TFK-1 cells receiving shRNAs specific to DNA polymerase θ (shPOLQ; #1 and #2) or LacZ (shLacZ) were treated with 1 μM GEM and 10 μM CDDP for 24 h before AZD0156 addition. The values (means ± SD) are from two data points, and are expressed as a percentage relative to that of the cells without AZD0156 treatment. The IC_50_ values for AZD0156 in the cell lines are shown in each panel. (**F**) The relative cell viability in 2.5 μM AZD0156 for 72 h. TFK-1 cells receiving shRNAs specific to DNA polymerase θ (shPOLQ; #1 and #2) or LacZ (shLacZ) were treated with 1 μM GEM and 10 μM CDDP for 24 h before AZD0156 addition. The values (means ± SD) are from three independent experiments, and are expressed as a percentage relative to that of the cells receiving shRNAs against LacZ. * *p* < 0.05 by Student’s *t*-test. (**G**) Western blots showing the level of H2AX Ser139 phosphorylation (γ-H2AX), PARP1, ATM Ser1981 phosphorylation, and total ATM in TGBC-24TKB cells receiving shRNAs specific to DNA polymerase θ (shPOLQ; #1 and #2) or LacZ (shLacZ). The cells were treated with 0.5 μM GEM and 5 μM CDDP for 24 h, and were then cultured in the absence (–) or presence (+) of 2.5 μM AZD0156 for another 4 h; α-tubulin was the loading control. (**H**) The cells described in panel G were fixed and stained with H2AX Ser139 phosphorylation (γ-H2AX) and nuclei. Scale bar = 10 µm. (**I**) The relative fluorescent intensity of γ-H2AX was measured for each field (*n* = 5) and expressed as the fold change relative to the level of the cells without chemotherapy drugs and AZD0156 treatment. The values (means ± SD) are from three independent experiments; * *p* < 0.05 by Student’s *t*-test.

Next, we investigated whether AZD0156 promoted a synergistic effect on cell survival in DNA polymerase θ-deficient BTC cells upon GEM- and CDDP-induced DNA damage. The knockdown of DNA polymerase θ increased AZD0156 sensitivity in TGBC-24TKB and TFK-1 cells upon GEM and CCDP treatment (Figure 6A,B,D,E). Furthermore, DNA polymerase θ depletion increased AZD0156-mediated cell death and enhanced cell death by about two-fold after GEM- and CDDP-induced DNA damage in TGBC-24TKB cells (Figure 6C). Consistently, DNA polymerase θ knockdown decreased AZD0156 IC_50_ and promoted greater cell death after GEM and CDDP treatment in TFK-1 cells (Figure 6F). To determine the DNA damage accumulation, γ-H2AX levels were detected by Western blot and immunofluorescence. In the control, TGBC-24TKB cells (shLacZ), chemotherapy drugs, or the ATM inhibitor AZD0156 increased H2AX phosphorylation and the cleavage of a few PARP1 proteins. After DNA polymerase θ depletion by shRNAs, the phosphorylated H2AX and cleaved PARP1 proteins were strengthened in the presence of chemotherapy drugs and AZD0156 (Figure 6G,H). Collectively, these data demonstrate that chemotherapy drug-induced DNA damage improves the significant effect of ATM inhibition and DNA polymerase θ depletion in BTC.

**Figure 7:**
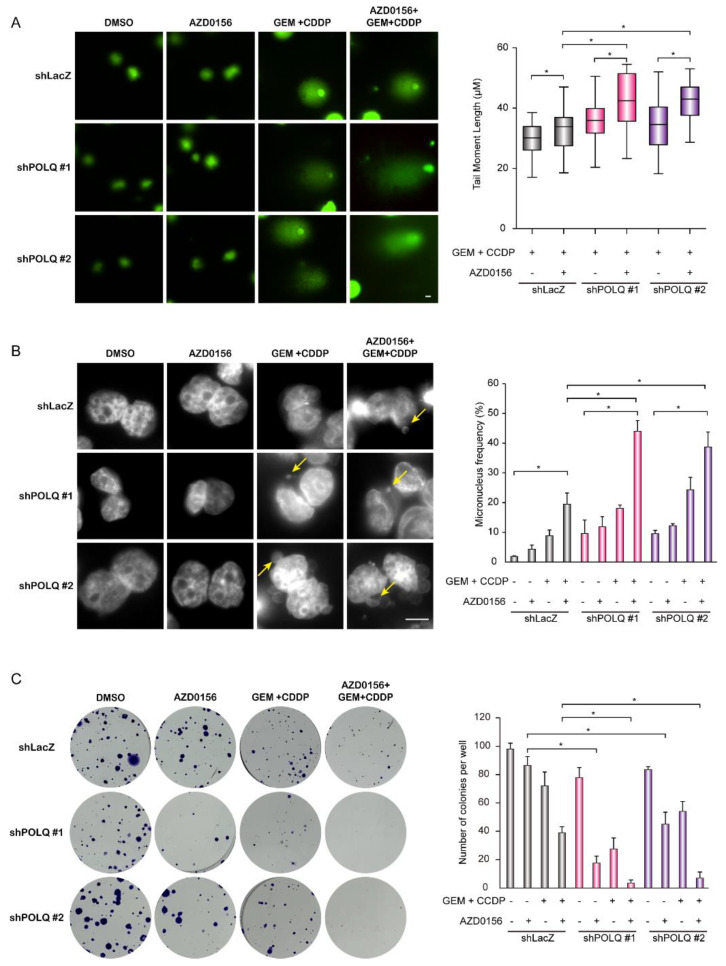
AZD0156 and DNA polymerase θ depletion leads to DNA damage accumulation and suppresses tumor growth. (**A**) The alkaline comet assay: TGBC-24TKB cells receiving shRNAs specific to DNA polymerase θ (shPOLQ) or LacZ (shLacZ) were treated with 0.5 μM GEM and 5 μM CDDP, followed by 5 μM AZD0156 for another 4 h before a comet assay. Left: the representative comet images are shown. Right, the quantitative data on the length of the tail movement. The values (means ± SD) are from two independent experiments; * *p* < 0.05 by Student’s *t*-test (*n* = 50). (**B**) Micronucleus frequency in TGBC-24TKB cells receiving shRNAs specific to DNA polymerase θ (shPOLQ) or LacZ (shLacZ). The cells were treated with 0.5 μM GEM and 5 μM CDDP for 24 h, and were then cultured in the absence or presence of 2.5 μM AZD0156 for another 24 h. Left: representative images of the micronucleus (arrows) are shown. Right, the quantitative data of the percentage of micronuclei. The values (means ± SD) are from two independent experiments; * *p* < 0.05 by Student’s *t*-test (*n* = 100). (**C**) The colony formation assay was performed in TGBC-24TKB cells receiving shRNAs specific to DNA polymerase θ (shPOLQ) or LacZ (shLacZ). The cells (400 cells/well) were seeded in a six-well plate for 14 days, cultured in the absence or presence of 0.5 μM GEM and 5 μM CCDP for 24 h, and then cultured in the absence or presence of 1 μM AZD0156 for another seven days. Left: representative images are shown. Right: the values (means ± SD) are from two independent experiments; * *p* < 0.05 by Student’s *t*-test.

To directly demonstrate DNA damage, alkaline comet assays were performed to detect DNA strand breaks. AZD0156 improved the comet tail movement in the control and DNA polymerase θ-depleted TGBC-24TKB cells. Moreover, the tail movement was significantly increased in TGBC-24TKB cells receiving DNA polymerase θ shRNAs in the presence of AZD0156 (Figure 7A). In the micronuclei, a combination of chemotherapy drugs and AZD0156 increased the micronucleus formation, and the knockdown of DNA polymerase θ markedly strengthened the frequency of micronucleus formation (Figure 7B). Finally, we used a colony formation assay to confirm the effect of AZD0156 and DNA polymerase θ depletion on cell survival. In the DNA polymerase θ knockdown cells, AZD0156 suppressed colony formation, with almost no colony formation observed upon chemotherapy drug-induced DNA damage (Figure 7C). Collectively, these data indicate that the ATM inhibitor AZD0156 and DNA polymerase θ depletion lead to DNA damage accumulation, resulting in cell death in BTC cells, particularly in the presence of cytotoxic agents.

## 4. Discussion

**Figure 8:**
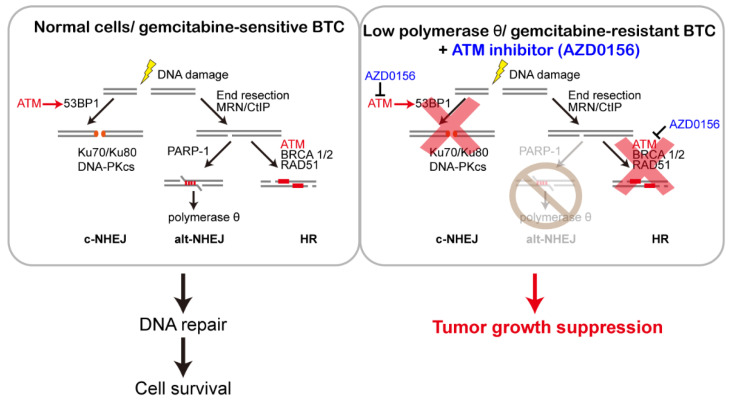
A diagram illustrating that the ATM inhibitor AZD0156 impairs tumor growth in gemcitabine-resistant or polymerase θ-deficient BTCs.

The mutations or over-expression of DDR genes are usually determined in various cancers. Thus, the target of the DDR pathway using a synthetic lethality approach may be a potential therapy for gemcitabine-resistant BTCs. In an in silico study, we found that the mRNA levels of the four genes (*RAD51*, *POLQ*, *FANCD2*, and *CHEK1*) in CCA were more than tenfold higher than those in normal tissue. Only *POLQ* expression was inversely correlated with overall survival. In gemcitabine-resistant BTC cells, *POLQ* mRNA expression was downregulated. Previous studies have indicated that three pathways are involved in DNA repair for DSBs [5]. Thus, we propose that in the case of low DNA polymerase θ expression or gemcitabine-insensitive/resistant BTC cells, the alt-NHEJ pathway is disrupted. Moreover, the ATM inhibitor (AZD0156) suppressed another two repair pathways, resulting in BTC tumor suppression due to synthetic lethality. We used photons, hydrogen peroxide, and chemotherapy drugs to induce DNA damage. Combined ATM inhibition by using AZD0156 and DNA polymerase θ depletion via shRNAs led to cell growth suppression and apoptosis in BTCs. All of these results indicate that AZD0156 suppresses cell growth in gemcitabine-resistant or DNA polymerase θ-deficient BTCs (Figure 8).

Targeting particular DNA repair pathways may be therapeutic options for various cancers. In 2005, PARP inhibitors were shown to suppress BRCA1/2-deficient tumors in breast cancer [20,21]. Since then, several studies have demonstrated the synthetic lethal effect on tumor growth in DNA damage responses in different cancers, such as lung cancer, breast cancer, pancreatic cancer, and ovarian cancer [22,23,24,25]. In BTCs, one report investigated the suppressive effect of PARP inhibitors on tumor growth in IDH (isocitrate dehydrogenase)-mutant cholangiocarcinomas [26]. In our study, we showed that the expression of *POLQ* is down-regulated in gemcitabine-resistant cells (Figure 1). The knockdown of DNA polymerase θ did not affect tumor cell growth, and a combination of ATM inhibition impaired cell growth (Figure 2). The synthetic lethal interaction of DNA polymerase θ and ATM was apparently not due to the knockdown system in our study. Nevertheless, a combination of photons, hydrogen peroxide, or chemotherapy drugs improved the synthetic lethal effect, increasing ATM inhibition and DNA polymerase θ depletion (Figure 4 and Figure 6). Thus, using an ATM inhibitor may be a therapeutic option for DNA polymerase θ-deficient tumors in gemcitabine-resistant BTCs.

ATM mutations are detected in 6.7% of BTC patients, and these mutations mostly reflect decreased ATM kinase activity [27]. The expression of DNA polymerase θ is a deficiency in most normal cells, and is upregulated in many cancers, such as BTC, ovarian cancer, and melanoma [10]. Moreover, the overexpression of DNA polymerase θ displayed a poor outcome in CCA patients (Figure 1C). In previous reports, the suppression of DNA polymerase θ increased the ionizing radiation sensitivity in bone marrow stromal cells [28]. DNA polymerase θ has been demonstrated to suppress homologous recombination (HR) repair by associating with RAD51 [10]. The knockout of DNA polymerase θ causes synthetic lethality in BRCA1 mutant cells and FANCD2-defective human cells [10,11]. Therefore, developing DNA polymerase θ inhibitors should be regarded as another therapeutic approach for tumors with ATM mutations or high DNA polymerase θ expression in BTC.

Apart from *ATM*, several DDR genes have been detected in BTC patients, such as *TP53*, *KMT2C,* and *ATR*. In a previous report, the inhibition of ATR by shRNAs or VE822 inhibitors led to the suppression of tumor growth in DNA polymerase θ-knockout breast cancer [29]. Thus, targeting ATR may be a potential strategy for DNA polymerase θ-deficient tumors in gemcitabine-resistant BTCs. Regarding *TP53*, the synthetic lethal interaction of p53 mutations and several candidate genes were identified using a computational approach [30], and some studies have demonstrated that the suppression of some genes results in a synthetic lethality in p53-defective cancers. UCN-01, a CHK1 inhibitor, represses ATR/ATM-mediated cell cycle checkpoint pathways in p53-defective cells, resulting in decreasing cell proliferation [31]. In acute myeloid leukemia, the combination of p53 activation and Bcl2 inhibition also causes a synthetic lethality [32]. Mutations of p53 were determined in about 21% of BTC patients. In BTC, a combination of the ATR inhibitor AZD6738 and CDDP decreases tumor growth in p53-mutated BTC cell lines (SNU-478 and SNU-869) [33]. Therefore, the development of potential targets for p53-mutated BTC patients should be further explored.

In conclusion, in this study, we demonstrated the role of ATM inhibition on tumor growth in gemcitabine-resistant and DNA polymerase θ-deficient BTC cells. DNA damage—induced by photons, superoxide, or chemotherapy drugs—can enhance the synthetic lethal effect caused by ATM inhibition and DNA polymerase θ depletion. In further clinical research, combinations of photons or chemotherapy drugs with the ATM inhibitor AZD0156 may achieve a better effect in repressing tumor growth for advanced BTC patients with low DNA polymerase θ expression.

## References

[1] Lamarca A., Barriuso J., McNamara M.G., Valle J.W. (2018). Biliary Tract Cancer: State of the Art and potential role of DNA Damage Repair. Cancer Treat. Rev..

[2] Silva V.W., Askan G., Daniel T.D., Lowery M., Klimstra D.S., Abou-Alfa G.K., Shia J. (2016). Biliary carcinomas: Pathology and the role of DNA mismatch repair deficiency. Chin. Clin. Oncol..

[3] Lord C.J., Ashworth A. (2012). The DNA damage response and cancer therapy. Nature.

[4] Liu Q., Lopez K., Murnane J., Humphrey T., Barcellos-Hoff M.H. (2019). Misrepair in Context: TGFbeta Regulation of DNA Repair. Front. Oncol..

[5] Ceccaldi R., Rondinelli B., D’Andrea A.D. (2016). Repair Pathway Choices and Consequences at the Double-Strand Break. Trends Cell Biol..

[6] Wood R.D., Doublie S. (2016). DNA polymerase theta (POLQ), double-strand break repair, and cancer. DNA Repair (Amst.).

[7] Lord C.J., Garrett M.D., Ashworth A. (2006). Targeting the double-strand DNA break repair pathway as a therapeutic strategy. Clin. Cancer Res..

[8] Harding S.M., Coackley C., Bristow R.G. (2011). ATM-dependent phosphorylation of 53BP1 in response to genomic stress in oxic and hypoxic cells. Radiother. Oncol..

[9] Gatei M., Scott S.P., Filippovitch I., Soronika N., Lavin M.F., Weber B., Khanna K.K. (2000). Role for ATM in DNA damage-induced phosphorylation of BRCA1. Cancer Res..

[10] Ceccaldi R., Liu J.C., Amunugama R., Hajdu I., Primack B., Petalcorin M.I., O’Connor K.W., Konstantinopoulos P.A., Elledge S.J., Boulton S.J. (2015). Homologous-recombination-deficient tumours are dependent on Poltheta-mediated repair. Nature.

[11] Mateos-Gomez P.A., Gong F., Nair N., Miller K.M., Lazzerini-Denchi E., Sfeir A. (2015). Mammalian polymerase theta promotes alternative NHEJ and suppresses recombination. Nature.

[12] Shima N., Munroe R.J., Schimenti J.C. (2004). The mouse genomic instability mutation *chaos1* is an allele of *Polq* that exhibits genetic interaction with *Atm*. Mol. Cell Biol..

[13] O’Neil N.J., Bailey M.L., Hieter P. (2017). Synthetic lethality and cancer. Nat. Rev. Genet..

[14] Hu X., Zhang Z. (2016). Understanding the Genetic Mechanisms of Cancer Drug Resistance Using Genomic Approaches. Trends Genet..

[15] Huang A., Garraway L.A., Ashworth A., Weber B. (2020). Synthetic lethality as an engine for cancer drug target discovery. Nat. Rev. Drug Discov..

[16] Liu S., Ge Y., Wang T., Edwards H., Ren Q., Jiang Y., Quan C., Wang G. (2017). Inhibition of ATR potentiates the cytotoxic effect of gemcitabine on pancreatic cancer cells through enhancement of DNA damage and abrogation of ribonucleotide reductase induction by gemcitabine. Oncol. Rep..

[17] Parsels L.A., Morgan M.A., Tanska D.M., Parsels J.D., Palmer B.D., Booth R.J., Denny W.A., Canman C.E., Kraker A.J., Lawrence T.S. (2009). Gemcitabine sensitization by checkpoint kinase 1 inhibition correlates with inhibition of a Rad51 DNA damage response in pancreatic cancer cells. Mol. Cancer Ther..

[18] Ewald B., Sampath D., Plunkett W. (2007). H2AX phosphorylation marks gemcitabine-induced stalled replication forks and their collapse upon S-phase checkpoint abrogation. Mol. Cancer Ther..

[19] Jayachandran G., Ueda K., Wang B., Roth J.A., Ji L. (2010). NPRL2 sensitizes human non-small cell lung cancer (NSCLC) cells to cisplatin treatment by regulating key components in the DNA repair pathway. PLoS ONE.

[20] Farmer H., McCabe N., Lord C.J., Tutt A.N., Johnson D.A., Richardson T.B., Santarosa M., Dillon K.J., Hickson I., Knights C. (2005). Targeting the DNA repair defect in BRCA mutant cells as a therapeutic strategy. Nature.

[21] Bryant H.E., Schultz N., Thomas H.D., Parker K.M., Flower D., Lopez E., Kyle S., Meuth M., Curtin N.J., Helleday T. (2005). Specific killing of BRCA2-deficient tumours with inhibitors of poly(ADP-ribose) polymerase. Nature.

[22] Lord C.J., Tutt A.N., Ashworth A. (2015). Synthetic lethality and cancer therapy: Lessons learned from the development of PARP inhibitors. Annu. Rev. Med..

[23] Turk A.A., Wisinski K.B. (2018). PARP inhibitors in breast cancer: Bringing synthetic lethality to the bedside. Cancer.

[24] Touat M., Sourisseau T., Dorvault N., Chabanon R.M., Garrido M., Morel D., Krastev D.B., Bigot L., Adam J., Frankum J.R. (2018). DNA repair deficiency sensitizes lung cancer cells to NAD+ biosynthesis blockade. J. Clin. Investig..

[25] Zhu H., Wei M., Xu J., Hua J., Liang C., Meng Q., Zhang Y., Liu J., Zhang B., Yu X. (2020). PARP inhibitors in pancreatic cancer: Molecular mechanisms and clinical applications. Mol. Cancer.

[26] Wang Y., Wild A.T., Turcan S., Wu W.H., Sigel C., Klimstra D.S., Ma X., Gong Y., Holland E.C., Huse J.T. (2020). Targeting therapeutic vulnerabilities with PARP inhibition and radiation in IDH-mutant gliomas and cholangiocarcinomas. Sci. Adv..

[27] Choi M., Kipps T., Kurzrock R. (2016). ATM Mutations in Cancer: Therapeutic Implications. Mol. Cancer Ther..

[28] Goff J.P., Shields D.S., Seki M., Choi S., Epperly M.W., Dixon T., Wang H., Bakkenist C.J., Dertinger S.D., Torous D.K. (2009). Lack of DNA polymerase theta (POLQ) radiosensitizes bone marrow stromal cells in vitro and increases reticulocyte micronuclei after total-body irradiation. Radiat. Res..

[29] Wang Z., Song Y., Li S., Kurian S., Xiang R., Chiba T., Wu X. (2019). DNA polymerase theta (POLQ) is important for repair of DNA double-strand breaks caused by fork collapse. J. Biol. Chem..

[30] Wang X., Simon R. (2013). Identification of potential synthetic lethal genes to p53 using a computational biology approach. BMC Med. Genom..

[31] Reinhardt H.C., Aslanian A.S., Lees J.A., Yaffe M.B. (2007). p53-deficient cells rely on ATM- and ATR-mediated checkpoint signaling through the p38MAPK/MK2 pathway for survival after DNA damage. Cancer Cell.

[32] Pan R., Ruvolo V., Mu H., Leverson J.D., Nichols G., Reed J.C., Konopleva M., Andreeff M. (2017). Synthetic Lethality of Combined Bcl-2 Inhibition and p53 Activation in AML: Mechanisms and Superior Antileukemic Efficacy. Cancer Cell.

[33] Nam A.R., Jin M.H., Park J.E., Bang J.H., Oh D.Y., Bang Y.J. (2019). Therapeutic Targeting of the DNA Damage Response Using an ATR Inhibitor in Biliary Tract Cancer. Cancer Res. Treat..

